# Ultrahigh-speed Si-integrated on-chip laser with tailored dynamic characteristics

**DOI:** 10.1038/srep38801

**Published:** 2016-12-09

**Authors:** Gyeong Cheol Park, Weiqi Xue, Molly Piels, Darko Zibar, Jesper Mørk, Elizaveta Semenova, Il-Sug Chung

**Affiliations:** 1 Department of Photonics Engineering (DTU Fotonik), Technical University of Denmark, DK-2800 Kgs. Lyngby, Denmark

## Abstract

For on-chip interconnects, an ideal light source should have an ultralow energy consumption per bandwidth (operating en-ergy) as well as sufficient output power for error-free detection. Nanocavity lasers have been considered the most ideal for smaller operating energy. However, they have a challenge in obtaining a sufficient output power. Here, as an alternative, we propose an ultrahigh-speed microcavity laser structure, based on a vertical cavity with a high-contrast grating (HCG) mirror for transverse magnetic (TM) polarisation. By using the TM HCG, a very small mode volume and an un-pumped compact optical feedback structure can be realised, which together tailor the frequency response function for achieving a very high speed at low injection currents. Furthermore, light can be emitted laterally into a Si waveguide. From an 1.54-*μ*m optically-pumped laser, a 3-dB frequency of 27 GHz was obtained at a pumping level corresponding to sub-mA. Using measured 3-dB frequen-cies and calculated equivalent currents, the modulation current efficiency factor (MCEF) is estimated to be 42.1 GHz/mA^1/2^, which is superior among microcavity lasers. This shows a high potential for a very high speed at low injection currents or avery small heat generation at high bitrates, which are highly desirable for both on-chip and off-chip applications.

For chip-level optical interconnects, a light source with an ultralow operating energy is a key element. The operating energy which measures the electric energy needed to generate an optical bit, should be around 10 fJ/bit for on-chip interconnects and around 100 fJ/bit for off-chip interconnects^1^. In addition, the bit error rate (BER) for the received signal should be lower than 10^−12^, which typically requires the received power be larger than several 10 s of microWatts^2^. We note that the forward error correction (FEC) as means to lower BER should be avoided, since it adds an operating energy of a few 100 s fJ/bit to a few pJ/bit^3^, which may dominate the operating energy budget. Therefore, an ideal on-chip laser should simultaneously satisfy the requirements on the operating energy as well as the output power.

To date, reducing the dimensions of a laser to the sub-micron scale has been considered the best way to realise an ideal on-chip laser, as nanocavity lasers can achieve a very small operating energy, e.g., 4.4 fJ/bit at 10 Gbit/s^4^, thanks to the small threshold current needed to make population inversion in a small active region. However, their output power is also low, e.g., 2.17 *μ*W at 4.4 fJ/bit^4^, requiring the FEC that should be avoided as discussed above. A microcavity laser with an output power more than 100 *μ*W can be an alternative if it can achieve a few 10 s Gbit/s speed at a sub-mA injection current. The capability of a laser achieving a high speed per injection current is measured by the MCEF, which is inversely proportional to the squareroot of the laser mode volume.

Among various microcavity structures, vertical cavity lasers (VCLs) are a good candidate, thanks to the small mode volume. For on-chip applications, a difficulty of emitting light from a VCL laterally into a Si waveguide can be solved by using a high-contrast grating (HCG) as a bottom mirror^5^,^6^,^7^. State-of-the art vertical-cavity surface-emitting lasers (VCSELs) show superior potentials for off-chip applications. An operating energy of 83 fJ/bit with an MCEF of 14 GHz/mA^1/2^ has been reported from an 850-nm VCSEL^2^. From an 1530-nm VCSEL, an FEC-free bitrate of 56-Gb/s has been demonstrated with an MCEF of about 8 GHz/mA^1/2^ 8. However, to meet on-chip requirements, a several times larger MCEF is required. For reference, the MCEF of a photonic crystal nanocavity laser is about 60 GHz/mA^1/2^ 4. Achieving such a high MCEF appears challenging in conventional VCSEL structures, since the cavity length of high-speed VCSELs is already squeezed to 1*λ* for a small mode volume.

In this Letter, we propose a novel way of tailoring the frequency response, based on the Si-integrated VCL (Si-VCL) structure with a TM HCG reflector. The tailoring consists of increasing the 3-dB frequency (*f*_3dB_) and flattening the shape of response function at low injection levels. The flattening of response spectrum allows for enhancing the modulation speed (B) at a given 3-dB frequency by diminishing the pulse-to-pulse interactions, called pattern effects. This tailoring enables to achieve a very high speed at low injection levels, resulting in a very high MCEF. The mirrors are designed to minimise the power penetration into mirrors, thus increasing the 3-dB frequency. An integrated lateral optical feedback structure enables to suppress the relaxation resonance as well as further increasing the 3-dB frequency. As an important feature, the feedback structure is compact and operates without carrier supply, thanks to the very slow lateral group velocity (group index, *n*_*g*_ = 151) in the HCG-based vertical cavity. This paper is organised as follows. Firstly, the laser structure is explained with elaborations on the mirror design and feedback structure. Then, static and dynamic characterisation results of a fabricated laser sample are presented with discussion on the impact of tailoring. The demonstrated 3-dB frequency of 27 GHz is, to our knowledge, the highest reported for any type of Si-integrated on-chip lasers and was limited by the characterisation set-up. The laser sample was characterised by using the optically pumping method. For analysis and comparison, the equivalent current is estimated from the measured optical input power (c.f. Methods). Finally, the application potentials of the proposed laser structure and possible issues for electrically pumped versions are discussed.

## Results

### Laser design

**Figure 1:**
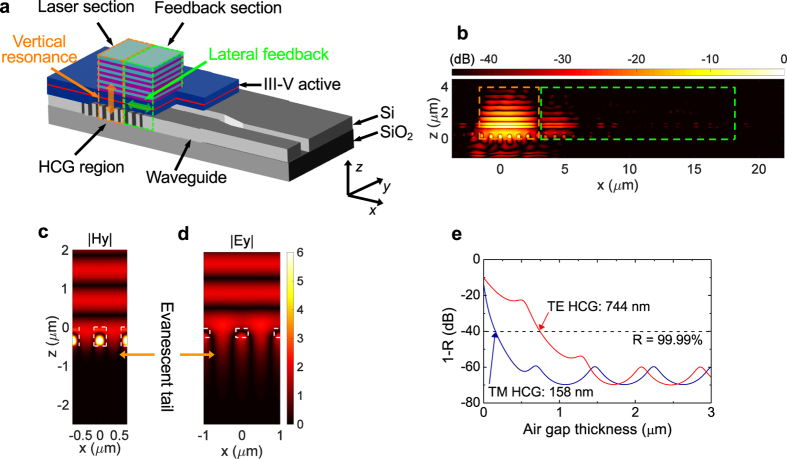
Design concepts. (**a**) Schematic view of the laser structure. Light is amplified vertically in the laser section (orange dotted box). Optical feedback is given to the laser section laterally from the feedback section (green dotted box). (**b**) Snapshot of field intensity (|*H*|^2^) profile obtained by the FDTD method. (**c,d**) Field profiles around a TM HCG (**c**) and a TE HCG (**d**) with a 1550-nm TM- and a TE-polarised incident field from the top, respectively. (**e**) Calculated (1 − *R*) of the III-V active/air gap/HCG, as a function of the air gap thickness.

As shown in Fig. 1a, the laser structure in the vertical direction (along the *z* axis) consists of a 6-pair Si/SiO _2_ distributed Bragg reflector (DBR), a 1*λ*-thick III-V active material including 7 InAlGaAs/InAlGaAs strained quantum wells (QWs), and an HCG region connected to a Si output waveguide. The active material is designed for a higher speed. The strained QWs with a large conduction band offset increase the differential gain, as well as reducing carrier leakage. The cladding layers are made of InP and efficiently spread heat so as to maintain a high peak gain value, as well as reducing the carrier leakage.

In the lateral direction (along the *x* axis) the laser structure is divided into the laser section designated by an orange dotted line and the feedback section designated by a green dotted line, as shown in Fig. 1a. Light is vertically amplified in the laser section and a fraction of it is laterally emitted into the feedback section, as shown in Fig. 1b (See also Supplementary Video 1). This lateral emission is reflected back into the laser section.

### Mirror design

Let us describe how the power penetration into mirrors is minimised for achieving a high 3-dB frequency. For light emission from the vertical cavity to the in-plane Si output waveguide, the bottom mirror should be a HCG reflector^5^,^6^,^7^. As shown in Fig. 1a, there is an air gap layer between the III-V active layer and the HCG layer, which is necessary since HCGs need to be surrounded by low refractive index materials in order to have a high reflectance^9^. The air gap should be as thin as possible to minimise the mode volume. The HCG design of this work allows the air gap to be as thin as 0.1*λ*. For comparison, the optical thickness of this low-index gap layer in prior HCG-based VCSELs ranges from 0.85*λ* to 1.2*λ*^10^,^11^. Let us discuss the background of this. HCGs can be classified into TM HCGs and TE HCGs: The TM HCGs are highly reflective to the electric field perpendicular to the grating lines, while the TE HCGs are, to the parallel field. As shown in Figs 1c and d, there is an evanescent field that extends from the grating layer into the low refractive index layers, i.e., air gap and buried oxide (BOX) layers. We have recently explained why the grating period is typically larger for TE HCGs than TM HCGs when both of them are designed for the same wavelength^12^. Since the evanescent tail length is inversely proportional to the grating period, TE HCGs typically have a longer evanescent tail, as compared in Figs 1c and d. Figure 1e shows that if the air gap layer is not thick enough, the evanescent tail couples to the III-V active layer, lowering the reflectance from the bottom mirror. The TM HCG design of this work only requires a 158-nm-thick air gap to reach 99.99-% reflectance, while an optimised TE HCG needs a 744-nm-thick air gap.

To further squeeze the mode volume, the power penetration into the top mirror is also minimised. Among CMOS compatible materials, Si/SiO_2_ with a high refractive index contrast of 1.47 is employed for the top dielectric DBR. This combination of the high-index-contrast dielectric DBR and TM HCG with a thin air gap results in a very high confinement factor of 12.2%, which is equivalent to a very small mode volume. For reference, a TE HCG based cavity has a confinement factor of 10.6% and a cavity based on GaAs/AlGaAs wafer-fused DBRs has ~4%, provided that the III-V active layer is identical.

### Feedback structure

Recently, it has been reported from DBR-based laterally-coupled-cavity VCSELs that an out-of-phase optical feedback can significantly increase the 3-dB frequency^13^,^14^. Therein, injection currents are several mA and the resonance peak intensity is not so large due to damping. This operating condition is typical for high-speed lasers for off-chip applications. However, in lasers for on-chip applications, the resonance peak is not sufficiently damped with small injection currents. When the resonance peak is large, the pattern effects make the opening of eye patterns smaller^15^. Thus, in on-chip lasers it is essential to suppress the resonance peak for achieving a high bitrate. As will be discussed in the characterisation result part, an optical feedback detuned from the out-of-phase condition can suppress the resonance peak as well as moderately increasing the 3-dB frequency.

Since the in-plane dispersion of HCG-based vertical cavities can be engineered^16^,^17^, the in-plane group velocity of the optical feedback can be feasibly controlled. In the Si-VCL samples of this work, the calculated group index in the *x* direction, *n*_g_ is as high as 151. As an even higher or smaller group index can be feasibly achieved by designing the HCG, the feedback section can be designed to target at a specific delay time. For reference, DBR-based cavities, the group index ranges 30 to 50^13^ and the extent of its engineering is limited. In addition, due to the slow group velocity in lateral direction, the finite size effect of a HCG^18^ can be effectively mitigated. As an important new feature compared to the prior approaches^14^, we emphasise that the integrated feedback section does not need to be pumped with carriers to avoid the absorption in QWs.

### Measurement results

**Figure 2:**
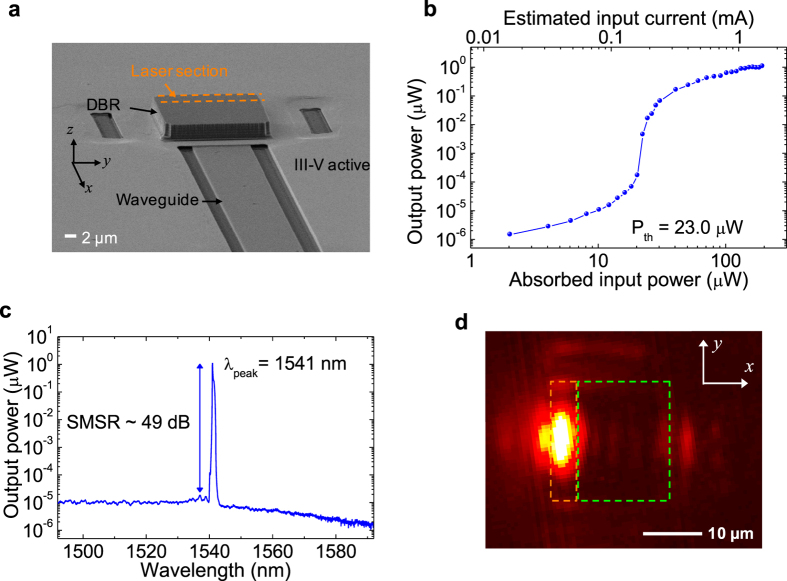
Experimental results of the fabricated Si-VCL. (**a**) Scanning electron microscope image of the fabricated Si-VCL. (**b**) Measured vertical light output as a function of input light. (**c**) Measured output spectrum at 8.4 times the threshold. (**d**) A infrared image taken from the top of the Si-VCL sample.

With these features, the sample shown in Fig. 2a was fabricated by using CMOS compatible processes (cf. Methods and Supplementary Fig. 1). Its static and dynamic properties were characterised by using the optical pumping method (see Supplementary Figs. 2 and 3). Figure 2b shows the output power as a function of input power (*P*_in_), defined as the absorbed power within the QW and barrier layers. The threshold input power, *P*_th_ is 23.0 *μ*W. The threshold current (*I*_th_) is conservatively estimated as corresponding to 0.18 ± 0.02 mA. It is explained in Methods how to convert optical input powers to equivalent currents. As shown in Fig. 2c, the lasing wavelength is 1541 nm and the side-mode suppression ratio (SMSR) is about 49 dB. Figure 2d shows an image of the light emission from the Si-VCL sample, taken from the top. The orange and green dotted boxes indicate the boundaries of the laser and feedback sections, respectively. The bright elliptical shape indicates the lasing mode within the laser section, while the dim periodic pattern on the right side of the laser mode is the standing wave in the feedback section.

**Figure 3:**
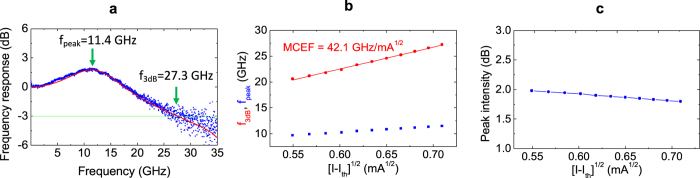
Dynamic characteristics of the Si-VCL. (**a**) The measured frequency response (blue) at *P*_in_ = 34.7 *μ*W and the simulated response at *I* = 0.68 mA (red). (**b**) The measured 3-dB frequency, *f*_3dB_ (red) and peak frequency, *f*_peak_, (blue) as a function of equivalent current, (*I* − *I*_th_)^1/2^, which is estimated from absorbed optical input powers (cf. the horizontal scales in Fig. 2b and Methods). (**c**) The measured peak intensity as a function of estimated (*I* − *I*_th_)^1/2^.

Figure 3a shows a measured small-signal response at an input power of 34.7 *μ*W, which is about 3 times of threshold, *P*_th_ and is estimated as corresponding to an injection current of 0.68 ± 0.08 mA. The 3-dB frequency (*f*_3dB_), peak frequency (*f*_peak_), and resonance peak are 27.3 GHz, 11.4 GHz, 1.7 dB, respectively. In conventional VCSELs, the ratio *f*_3dB_/*f*_peak_ is ~1.55^19^ and the resonance peak intensity is usually large, e.g., ~3 dB around 3 times of threshold^20^,^21^, while the ratio and peak intensity are ~2.4 and 1.7 dB in the laser of this study, respectively, as shown in Fig. 3a. Similar trends are observed also at different low injection levels, as shown in Figs 3b and c. It appears that an increased damping due to the optical feedback suppresses the relaxation resonance, decreasing the resonance peak intensity and moving the peak position to a lower frequency. The red curve in Fig. 3a is a fitting curve, based on rate equation models with delayed feedback (cf. Supplementary Information). The fitting curve shows that there is an additional weak resonance peak around *f* = 33 GHz, which makes the decay rate beyond the resonance frequency slower, i.e., −13 to −15 dB/decade. In conventional microcavity lasers, this decay rate is about −40 dB/decade. As a result, the 3-dB frequency can be larger than the case without optical feedback.

Figure 3b shows the *f*_3dB_ and *f*_peak_ as a function of (*I* − *I*_th_)^1/2^. As discussed above, *f*_3dB_/*f*_peak_ is considerably larger than 1.55 over various injection levels. The MCEF which is the slope of the *f*_3dB_ graph, is 42.1 GHz/mA^1/2^. It is noteworthy that this value is several times larger than those of conventional DBR-based VCSELs^2^,^8^ and is comparable to that of the PhC laser^4^. This shows that the 3-dB frequency is significantly enhanced by the small mode volume and optical feedback.

## Discussion

The high MCEF of the proposed laser structure has important potentials. At low injection levels, it may enable to achieve a low operating energy, which is desirable for on-chip lasers. At high injection levels, it may greatly reduce the heat generation, which is dominated by Joule heating around the active region. For example, a laser with a 3 times larger MCEF than a reference laser generates 81-times smaller heat (∝ *I*^4^) to achieve the same speed as the reference laser. This much less heat generation may significantly improve the lifetime reliability of microcavity lasers, which is of critical importance in optical modules for datacentre applications, especially at high bitrates.

In order to realise similar high-speed characteristics in an electric injection version of the proposed laser structure, small parasitics, efficient carrier injection, and efficient heat dissipation are essential. To avoid the high intrinsic speed being limited by parasitics, the series resistance and capacitance of the laser structure should be minimised. A tunnel junction can be used to lower the resistance, which enables to use low-resistance n-type InP layers for both contacts^22^. For metal contacts, the intra-cavity contact scheme is advantageous for minimising the capacitance. Proton implantation can be used to further lower the capacitance, as well as forming a current aperture^23^, which can effectively confine carriers only within the laser section. The regrowth after wafer bonding^24^, substrate removal, and patterning of the tunnel junction layer^22^ is another possibility for forming a current aperture. The heat mostly generated in the III-V active region can be effectively removed by forming a metallic heat spreader or channel^5^,^23^. In this way, a small thermal resistance is achievable and CW operation above 70 °C is possible.

The differential quantum efficiency (DQE) of the laser of this work is about 3.6%. This small value results from the top mirror reflectivity of 99.97%. By decreasing the reflectivity to 99.7%, the DQE can be increased to 28.6% while maintaining the same dynamic characteristics.

In conclusion, we have shown that a microcavity laser with a very high speed at low injection levels can be realised by tailoring the frequency response of the laser structure, based on the HCG-based vertical cavity platform. This laser platform can be flexibly designed for on-chip interconnects with a very small operating energy, as well as for large data centre or high-performance computer applications with a potential of long-time reliabiblity. We believe the concept of engineering the frequency response is applicable to other laser platforms including nano lasers and other material systems, e.g., GaAs.

## Methods

### Simulation

Reflection properties of HCGs and mode files shown in Fig. 1c to e are obtained by using a two-dimensional (2D) rigorous coupled wave analysis (RCWA) method simulator. A 2D finite-difference time-domain (FDTD) method simulator was used to obtain the mode profile shown in Fig. 1c and absorption efficiency (*η*_abs_). The absorption efficiency is the fraction of absorbed power in the QW and barrier layers over the power delivered onto the laser sample surface and was estimated to be 9.1%. Based on the mode profiles obtained by FDTD simulations, confinement factor was estimated by using a time-averaged method^25^.

### Fabrication

The Si-VCL in Fig. 2a was fabricated by using three main fabrication processes: (1) electron-beam lithography for patterning the HCG region and the output waveguide in the Si layer of a silicon-on-insulator (SOI) wafer, (2) a direct wafer bonding process at 300 °C for forming heterogeneous structure of the III-V active layer and the Si HCG, and (3) a deposition of a 6-period Si/SiO_2_ DBR and subsequent patterning. In addition, a selective wet etch was conducted to form the air gap layer between the III-V active and Si HCG layers. The air gap area is larger than the laser section area. The SOI wafer includes a 500 nm-thick Si layer and a 3 *μ*m-thick buried oxide layer (see also Supplementary Fig. 1).

### Characterisation

A set-up used for static characterisation is shown in Supplementary Fig. 2. The uncooled laser sample was optically pumped by using a 980-nm diode laser in pulsed operation mode with a 10-% duty cycle (*f*_pmp_) and a 5-MHz repetition rate. The pulsed operation was required, due to the absorption of the pumping light in the Si layers. The pumping light was delivered to the laser sample through alignment optics including a × 50 near-infrared objective lens. The power transmittance of the alignment optics, *T*_*a*_ is between 10.5 and 13.4%, depending on the alignment of the setup. The vertical output through the DBR was collected by the same objective lens, coupled through a fibre coupler, and sent to an optical spectrum analyser. The in-plane output was collected by a cleaved multi-mode fibre directly abutting the end of the Si waveguide. The light input power (*P*_in_) is estimated as *P*_in_ = *P*_pmp_ × *T*_a_ × *η*_abs_, where *P*_pmp_ is the power from the pump laser. The current for a continuous wave case (*I*) is estimated as *I* = *P*_in_/(*qE*_980_*f*_pmp_), where *E*_980_ is the photon energy at the pumping wavelength and *q* is the electron charge.

The set-up used for characterising small-signal frequency response is shown in Supplementary Fig. 3. The 980-nm diode laser was operated with a 4-% duty cycle and a 5-MHz repetition rate. The laser output was sent to a Mach-Zehnder modulator controlled by a network analyser with −5-dBm input power to the modulator. The modulated output was delivered to the laser sample by the alignment optics. The output from the laser sample was collected by an objective lens, fed into a fibre coupler, and amplified by an erbium-doped fibre amplifier (EDFA). After blocking the pump laser wavelength using an optical pass-band filter, the amplified signal was detected by a high-speed photo-detector connected to the 40-GHz HP network analyser. Lastly, the responses of the Mach-Zehnder modulator, the photo-detector, and the network analyser were subtracted from the measured S_21_ spectrum.

## Additional Information

**How to cite this article**: Park, G. C. *et al*. Ultrahigh-speed Si-integrated on-chip laser with tailored dynamic characteristics. *Sci. Rep.* **6**, 38801; doi: 10.1038/srep38801 (2016).

**Publisher's note:** Springer Nature remains neutral with regard to jurisdictional claims in published maps and institutional affiliations.

## Supplementary Material

Supplementary Video

Supplementary Information
