# Supplementary material for: Ultrahigh-speed Si-integrated on-chip laser with tailored dynamic characteristics
Source: Sci Rep. 2016 Dec 9;6:38801. doi: 10.1038/srep38801 (PMC5146957; doi:10.1038/srep38801)
Supplement: Supplementary Information [file srep38801-s2.pdf]

<u>Supplementary Information</u>

Ultrahigh-speed Si-integrated on-chip laser with tailored dynamic characteristics

Gyeong Cheol Park, Weiqi Xue, Molly Piels, Darko Zibar,
Jesper Mørk, Elizaveta Semenova, and Il-Sug Chung*

Department of Photonics Engineering (DTU Fotonik),
Technical University of Denmark, DK-2800 Kgs. Lyngby, Denmark.

* e-mail: ilch@fotonik.dtu.dk

I. FABRICATION PROCESS AND CHARACTERISATION SET-UPS

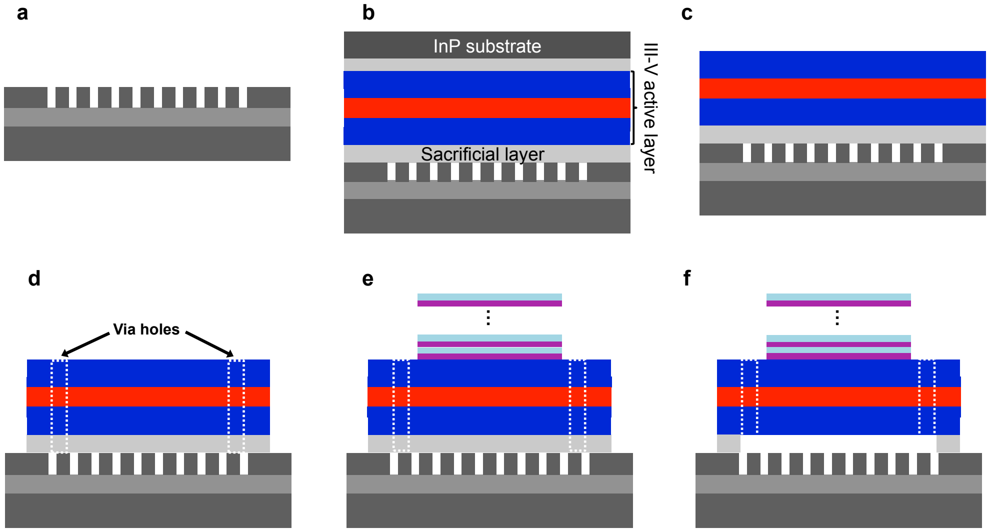

Supplementary Figure 1. **Fabrication procedure of the Si-VCL.** a) The HCG and the Si waveguide pattern (not shown here) were formed in the Si layer of a silicon-on-insulator (SOI) wafer by using electron-beam lithography and dry etching. b) The SOI and the III-V sample were rigorously cleaned using a standard RCA-1 (H_2O:H_2SO_4:H_2O_2) cleaning process. The III-V sample was put upside down on the SOI sample manually and then the bonded sample was put on the bonding machine with applying force and temperature. c) The InP substrate and the thin InGaAs etch stop layers were removed using wet etchant in sequence. d) The mesa pattern for the III-V layer was defined by using a SiN_x hard mask and reactive ion etching (RIE) process. At the same time, two via holes were defined for formation of an air gap above the HCG. e) 6-pair Si/SiO_2 DBR layer were deposited using a dielectric evaporator. Then, the square DBR was defined by using a AZ 4562E photo-resist hard mask and inductively coupled plasma reactive ion etching (ICP-RIE). f) To introduce the air gap, the sacrificial layer were subsequently removed through the two via holes by using wet etchant.

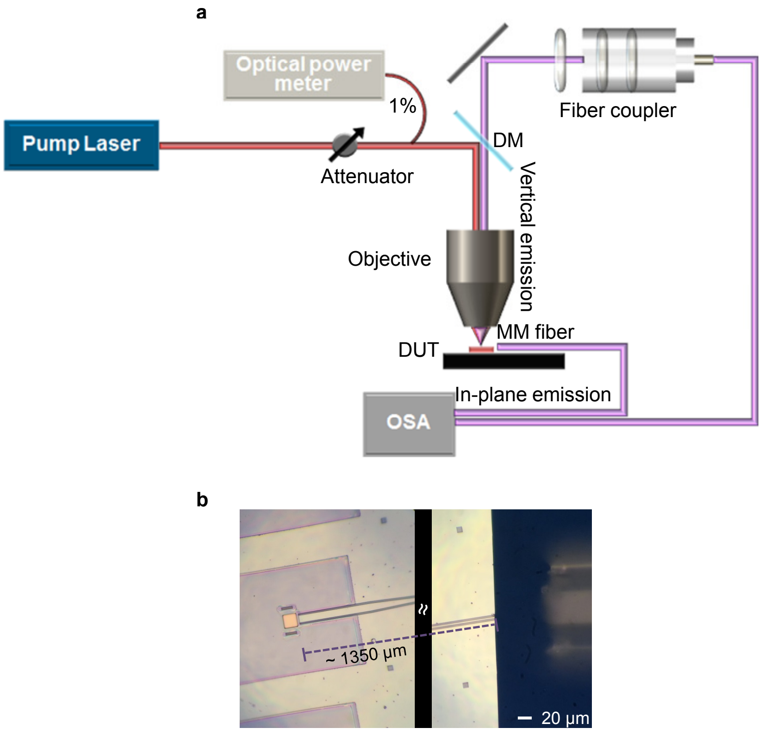

Supplementary Figure 2. **Static characterisation set-up.** a) Schematic illustration of the measurement set-up for the vertical and in-plane static characterisation. b) Microscopic image of the fabricated Si-VCL sample seen from the top and the multi-mode fibre near the end of the Si waveguide for the in-plane characterization. DM: Dichroic mirror. MM fibre: Multimode fibre, DUT: Device under test. OSA: Optical spectrum analyser.

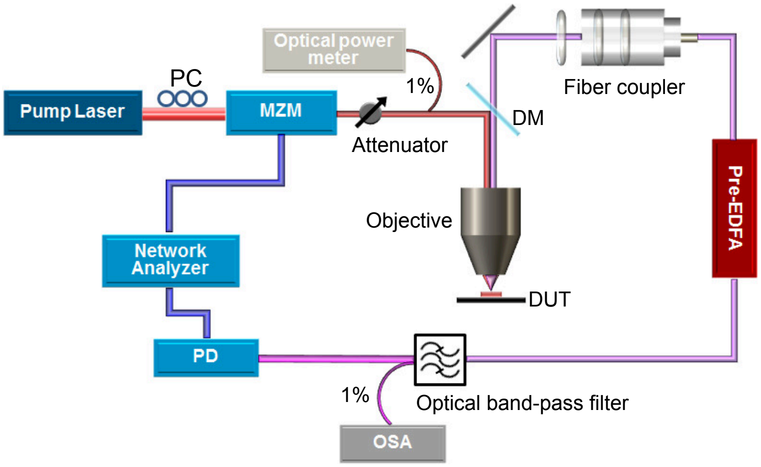

Supplementary Figure 3. **Dynamic characterisation set-up.** Schematic illustration of the measurement set-up for the dynamic characterisation. PC: Polarisation controller. MZM: Mach-Zehnder modulator. PD: Photo-detector.

II. FITTING RATE EQUATION MODEL

To analyse the eye diagram characteristics of the Si-VCL, the standard density rate equations [1] were used with an optical feedback term added [2, 3], assuming that multiple reflections in the laser section is negligible. The feedback term is the last term in Eq. 1b.

$$\frac{dN(t)}{dt} = \frac{\eta_i I(t)}{qV} - \frac{N(t)}{\tau_N} - v_g g N_P \tag{1a}$$

$$\frac{dN_p(t)}{dt} = \left[\Gamma v_g g - \frac{1}{\tau_p}\right] N_p(t) + \frac{\Gamma}{\tau_N'} N(t) + 2K_c \cos\theta \times \sqrt{N_p(t)N_p(t-\tau)} \tag{1b}$$

where K_c is the feedback rate, θ is the phase of the optical feedback relative to the field of the laser cavity, τ is the round-trip time of the optical feedback, N is carrier density, N_p is photon density, I is injection current, η_i is injection efficiency, q is electron charge, τ_N is carrier lifetime, v_g is group velocity, τ_p is photon lifetime, τ_N' is lifetime of carriers that radiate spontaneous emission into the lasing mode, and Γ is confinement factor.

The gain function, g is given by,

$$g(N, N_p, T) = \frac{1}{1+\epsilon N_p} g_0(N, T) \tag{2}$$

where the nonlinear gain compression factor, ϵ is 1.5×10^{-17}cm^3, and g_0 is obtained by using the 6 band k·p method as a function of carrier density, N and temperature, T. An example gain spectrum is presented in Supplementary Fig. 4, of which the peak wavelength agrees well with that of the measured photoluminescence (PL) spectrum.

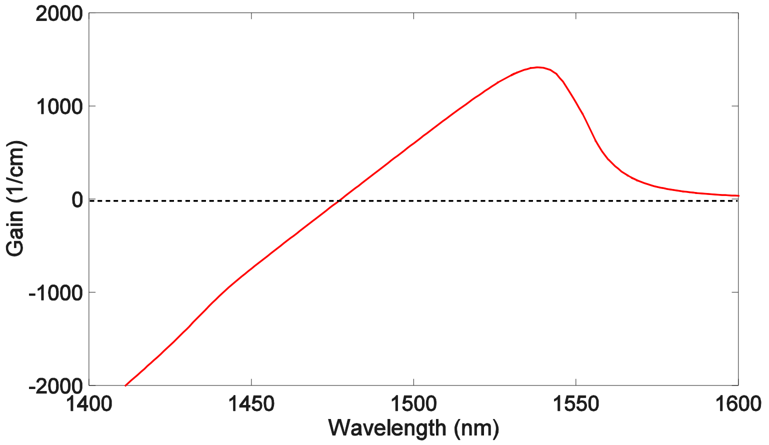

Supplementary Figure 4. **Gain model.** The gain spectra is evaluated at $N = 2.04 \times 10^{18}$ cm^{-3}, $N_p = 2.00 \times 10^{15}$ cm^{-3}, and $T = 23$°C.

The feedback related parameters (K_c, θ, and τ), the internal loss, α_i were determined by fitting the measured frequency response spectrum at an input power of 34.7 μW (blue curve in Fig. 3b), with the frequency response function, $|H|^2$ derived from the rate equations. The injection current, I_0 and injection efficiency, η_i are assumed to be 0.68 mA and 0.95, respectively. The frequency response function, H is given by,

$$H(\omega) \equiv \frac{N_{p1}}{I_1} = \frac{\eta_i}{qV} \frac{\gamma_{PN}}{\omega_R^2 - \omega^2 + i\omega\gamma}, \qquad (\mathbf{3})$$

where

$$\omega_R^2 = \gamma_{NP}\gamma_{PN} + \gamma_{NN}\gamma_{PP}, \qquad (\mathbf{4}\text{a})$$

$$\gamma = \gamma_{NN} + \gamma_{PP}, \qquad (\mathbf{4}\text{b})$$

$$\gamma_{NN} = 1/\tau_{\Delta N} + v_g a N_{p0}, \qquad (\mathbf{4}\text{c})$$

$$\gamma_{NP} = v_g g - v_g a_p N_{p0}, \qquad (\mathbf{4}\text{d})$$

$$\gamma_{PN} = \Gamma v_g a N_{p0} + \Gamma/\tau'_{\Delta N}, \qquad (\mathbf{4}\text{e})$$

$$\gamma_{PP} = -\Gamma v_g g + \Gamma v_g a_p N_{p1} N_{p0} + 1/\tau_p - K_c \cos\theta(1 + e^{-i\omega\tau}), \qquad (\mathbf{4}\text{f})$$

N_{p1} is the small signal amplitude of photon density, I_1, the small signal amplitude of injection current, N_0, the DC component of carrier density, N_{p0}, the DC component of photon density, $\tau_{\Delta N}$, differential carrier lifetime, $\tau'_{\Delta N}$, differential lifetime of carriers, a, differential gain, and a_p, differential gain depending on photon density. The fitting result, the red curve in Fig. 3b, shows a good agreement with the measured spectrum. This determined K_c, τ, θ and ϵ to be 150 GHz, 19.8 ps, 1.44π and $3 \times 10^{-17}\text{cm}^3$, respectively.

[1] Coldren, L. A., Corzine, S. W. & Mashanovitch, M. L. Diode lasers and photonic integrated circuits (Wiley, 2012).

[2] Lang, R. & Kobayashi, K. External optical feedback effects on semiconductor injection laser properties. *IEEE J. Quantum Electron.* **QE–16**, 347–355 (1980).

[3] Dalir, H. & Koyama, F. Bandwidth enhancement of single-mode VCSEL with lateral optical feedback of slow light. *IEICE Electronics Express* **8**, 1075–1081 (2011).

[Supplementary Video legend] Vertical cavity mode with optical feedback in lateral direction. Lasing mode resonates in the vertical direction while optical feedback is emitted and coupled back in the lateral direction. This video was obtained by using FDTD method.
